# Supplementary figures and images for: Cell2Spatial is a computational framework that maps single cells to spatial transcriptomic spots to reconstruct tissue architecture
Source: PLoS Biol. 2025 Nov 17;23(11):e3003477. doi: 10.1371/journal.pbio.3003477 (PMC12638031; doi:10.1371/journal.pbio.3003477)

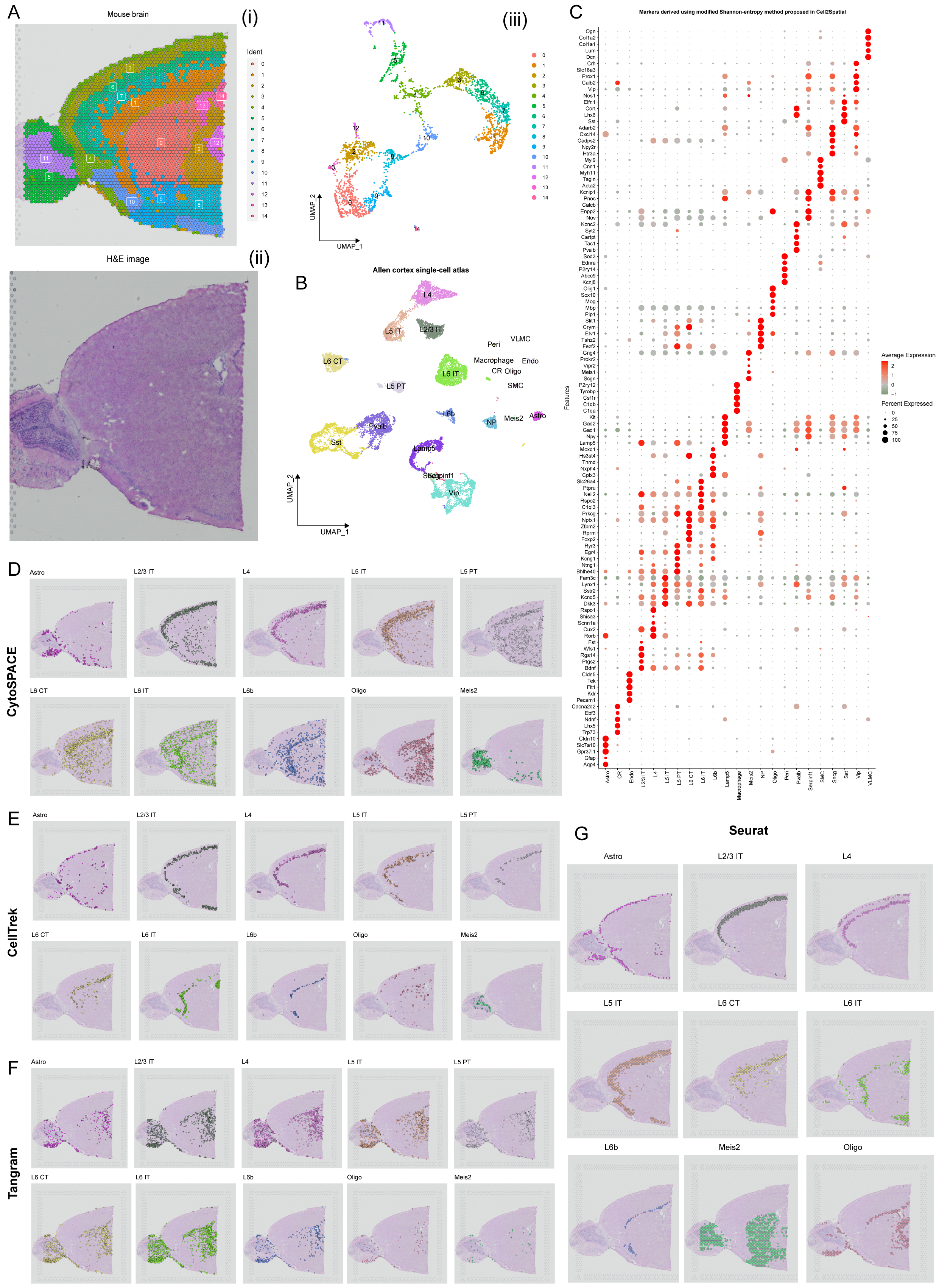

Supplement: S1 Fig — (A) (i) Clustering of spots from mouse brain spatial transcriptomics (ST) data, with clusters color-coded. (ii) Hematoxylin and eosin (H&E) staining image of mouse brain. (iii) Uniform Manifold Approximation and Projection (UMAP) showing clusters of spots inferred using Seurat [18] common processes. (B) UMAP plot showing the single-cell atlas of mouse brain. Each dot represents an individual cell. Cell types are marked by color codes. (C) Bubble plot showing the expression of five representative cell-type–specific marker genes selected from the top 30 overexpressed genes, as inferred by Cell2Spatial. Color intensity reflects the expression level of each gene in each cell type, while dot size corresponds to the proportion of cells expressing the gene. (D–G) Distribution of selected cell types with distinct spatial locations in mouse brain tissue, depicted using various mapping tools: (D) CytoSPACE, (E) CellTrek, (F) Tangram, and (G) Seurat. Each dot represents an individual cell. The data underlying this figure can be found at https://doi.org/10.5281/zenodo.17212677. (TIF) [file pbio.3003477.s001.tif]

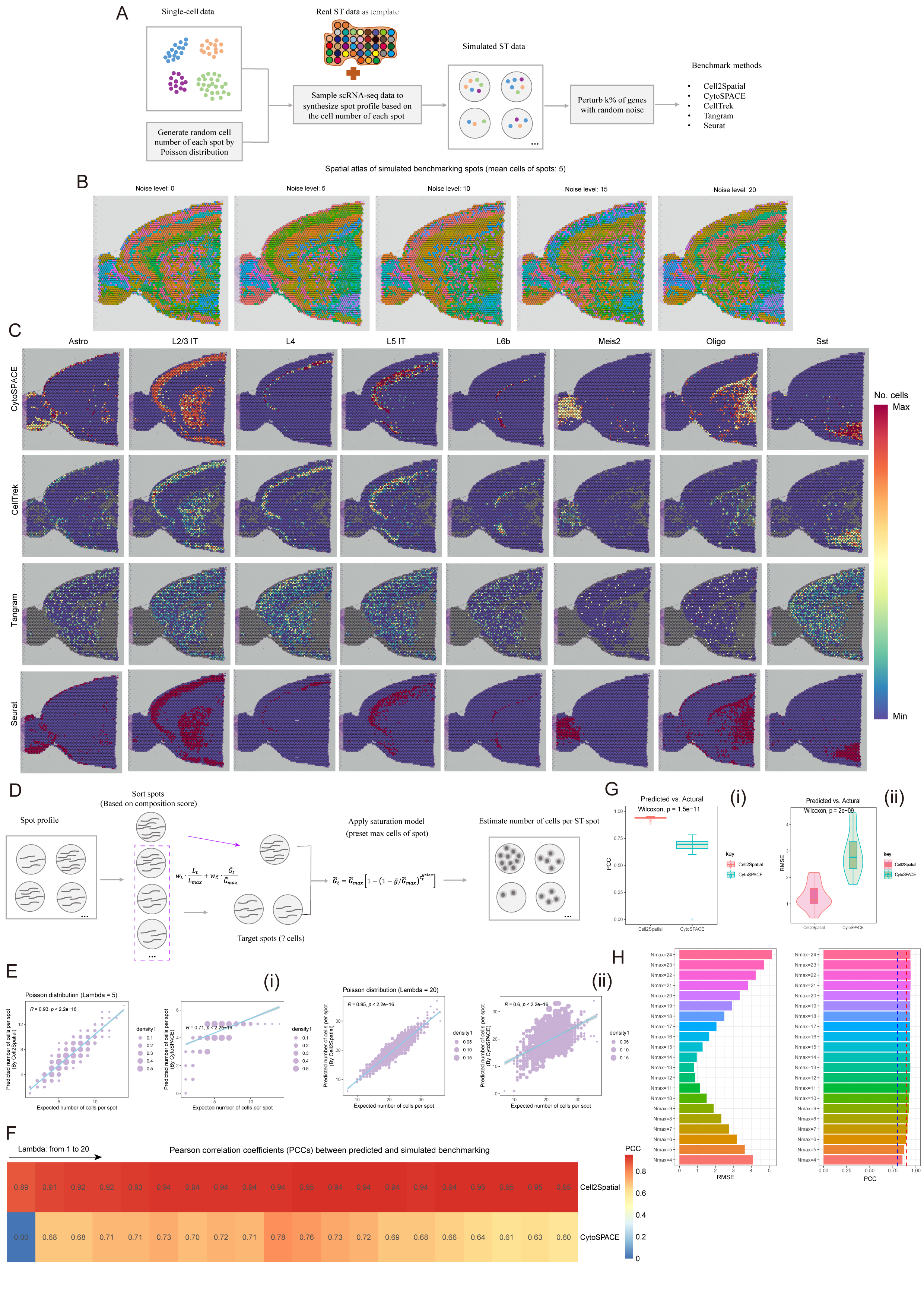

Supplement: S2 Fig — (A) Synthetic data generation strategy: This strategy comprises three main steps: (i) Randomly sampling cell counts for each spot based on a Poisson distribution, (ii) Assessing single-cell (SC) and spot similarity, and (iii) Aggregating the expression of nearby single cells based on the cell counts determined in step (i). To introduce variability, k% of genes are randomly perturbed with noise, resulting in synthetic ST data with diverse noise profiles (see “Materials and methods”). (B) Clustering of synthetic spots with varying noise levels on mouse brain tissue section. (C) Spatial heat maps showing the performance of publicly available mapping tools (CytoSPACE, CellTrek, Tangram, and Seurat) for aligning SC data (with 5% added noise) to spatial spots in ST datasets simulated with five cells on average (see “Materials and methods”). To enhance clarity, we have presented only cell types with prominent spatial structures. The color intensity of each spot corresponds to the number of single cells assigned. (D) Strategy for estimating the number of cells in spots (see “Materials and methods”). (E) Concordance between the predicted and expected number of cells based on the synthetic mouse brain ST data, with dot size indicating the density of spots sharing the same predicted cell count. (i) Lambda = 5; (ii) Lambda = 20. Cell2Spatial (left panel); CytoSPACE (right panel). (F) Heat map showing the Pearson correlation coefficients (PCCs) between predicted and simulated benchmarking data for both Cell2Spatial and CytoSPACE. Lambda values ranging from 1 to 20 were used for random sampling the number of cells following a Poisson distribution. (G) Box and violin plots displaying the distributions of PCCs (i) and root mean square errors (RMSEs) (ii) between predicted and actual counts in spots for Cell2Spatial and CytoSPACE. P-values were determined using the Wilcoxon tests. (H) Bar plot showing the influence of the preset maximum cell count per spot on prediction accuracy in [file pbio.3003477.s002.tif]

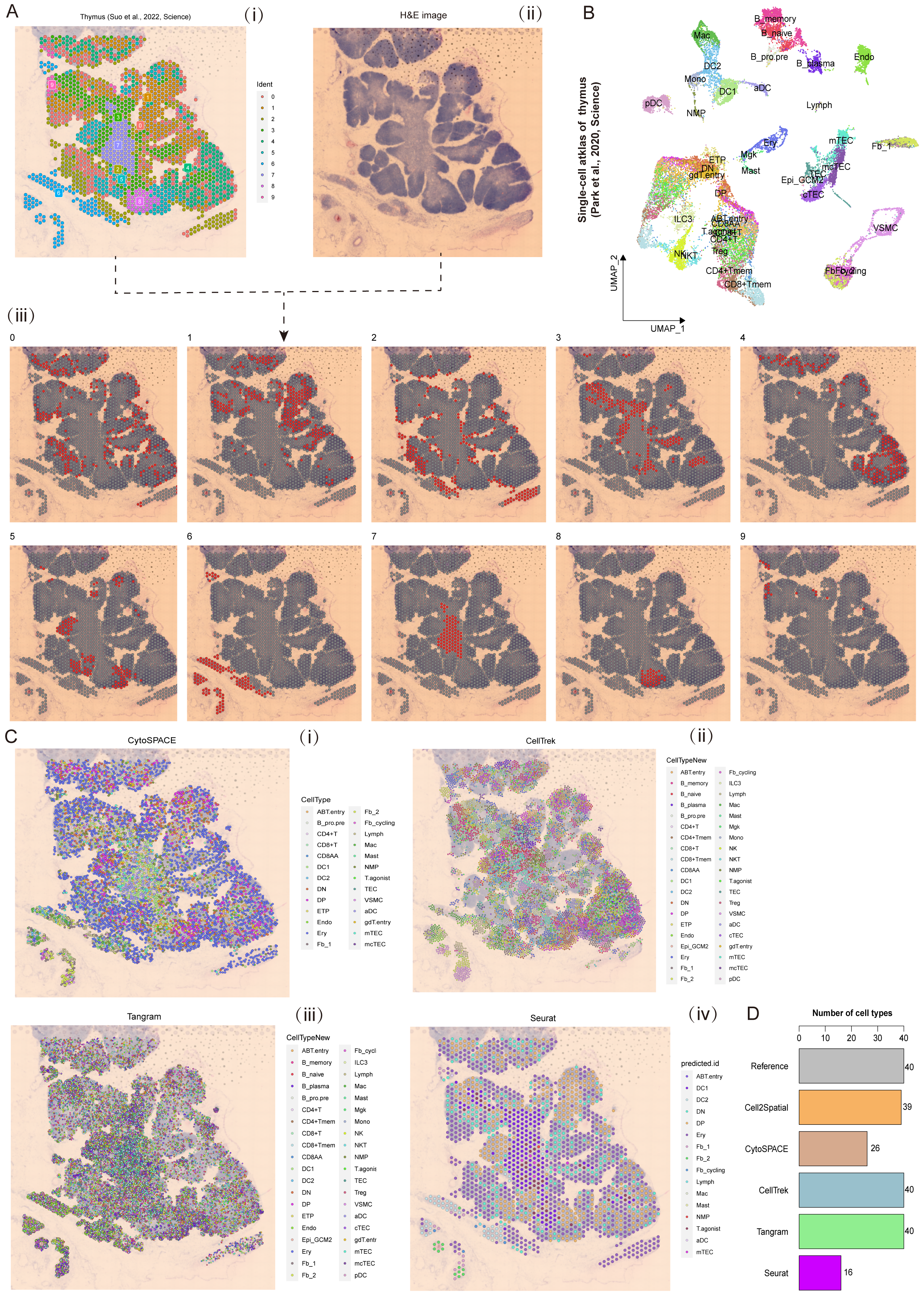

Supplement: S3 Fig — (A) (i) Clustering of spots from human thymus spatial transcriptomics (ST) data, with clusters color-coded. (ii) Hematoxylin and eosin (H&E) staining image of the thymus. (iii) Spatial location of each cluster on thymus ST section, highlighted by red color. (B) Uniform Manifold Approximation and Projection (UMAP) plot showing the single-cell atlas of human thymus. Each dot represents an individual cell, and cell types are marked by colors. (C) Spatial architectures of human thymus reconstructed using CytoSPACE (i), CellTrek (ii), Tangram (iii), and Seurat (iv). Each dot represents an individual cell. Cell types are marked by color codes. (D) Bar plot showing the number of cell types effectively mapped to spatial locations. “Reference” represents the total number of cell types in the single-cell atlas of the human thymus. The underlying data for this figure can be found at https://zenodo.org/records/17212677. (TIF) [file pbio.3003477.s003.tif]

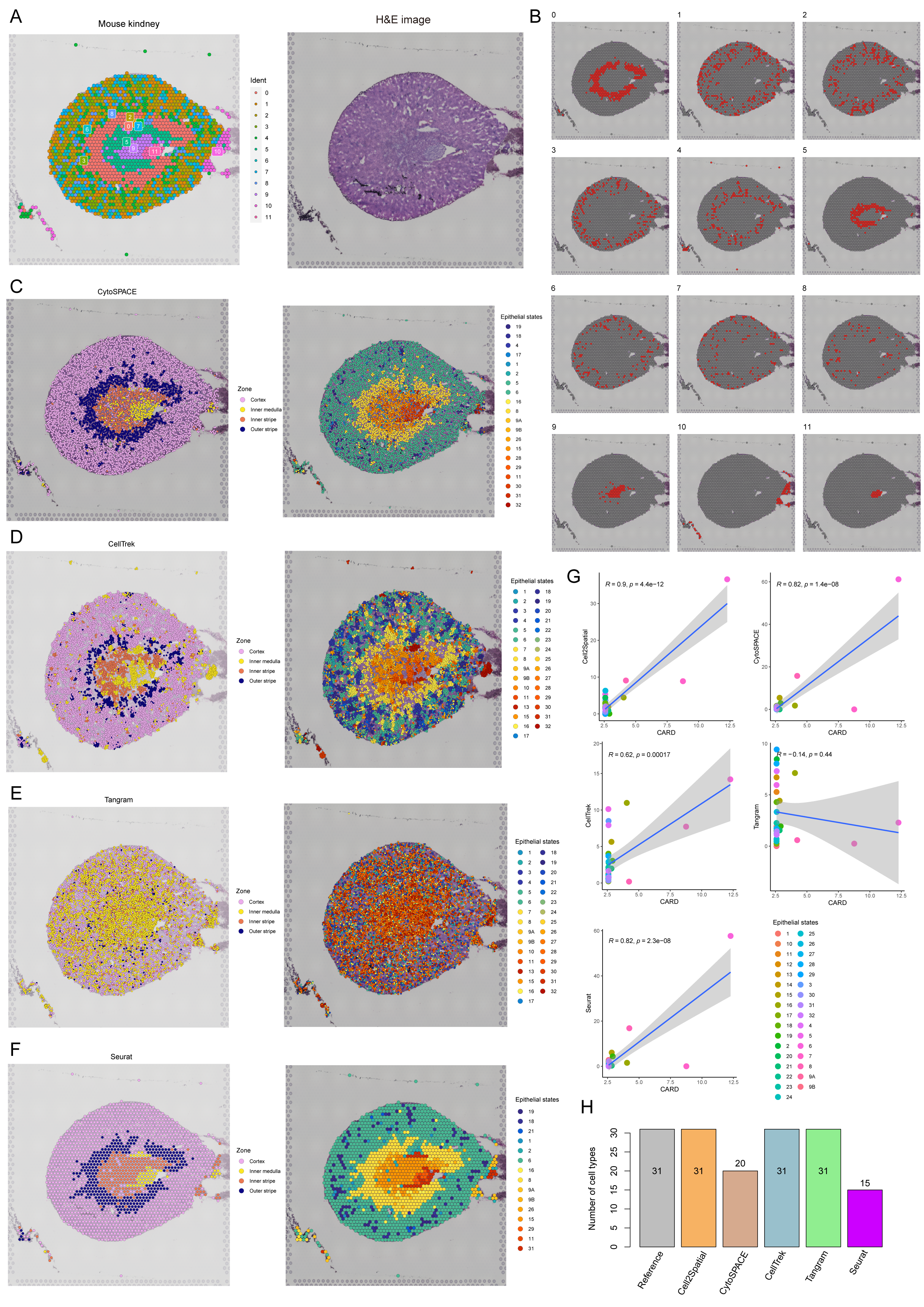

Supplement: S4 Fig — (A) Clustering spots of mouse kidney spatial transcriptomics (ST) data. Clusters are marked by color codes (left). Hematoxylin and eosin (H&E) staining of the corresponding kidney section (right). (B) Spots within each cluster on the mouse kidney ST section highlighted in red. (C–F) Mapping of epithelial cell transcriptomes from the mouse kidney single-cell atlas onto spatial spots of the corresponding ST section. Left: reconstructed spatial architectures with cells displayed using jitter within assigned spots. Right: the same representations with cells colored according to their known distance from the inner medulla. (C) CytoSPACE; (D) CellTrek; (E) Tangram; (F) Seurat. (G) Scatterplot showing the consistency between the cellular proportions in spatial architectures reconstructed with various mapping tools and cellular compositions predicted by the CARD spatial deconvolution tool [11]. The blue line denotes the linear fit, and the shaded area represents the 95% confidence interval. Different colors of points indicate distinct cell types. “R” represents the Pearson correlation coefficient (PCC). P-values were obtained using two-sided t-tests. (H) Bar plot showing the number of cell types effectively mapped to spatial locations. “Reference” represents the total number of cell types in the single-cell atlas of the mouse kidney. The underlying data for this figure can be found at https://zenodo.org/records/17212677. (TIF) [file pbio.3003477.s004.tif]

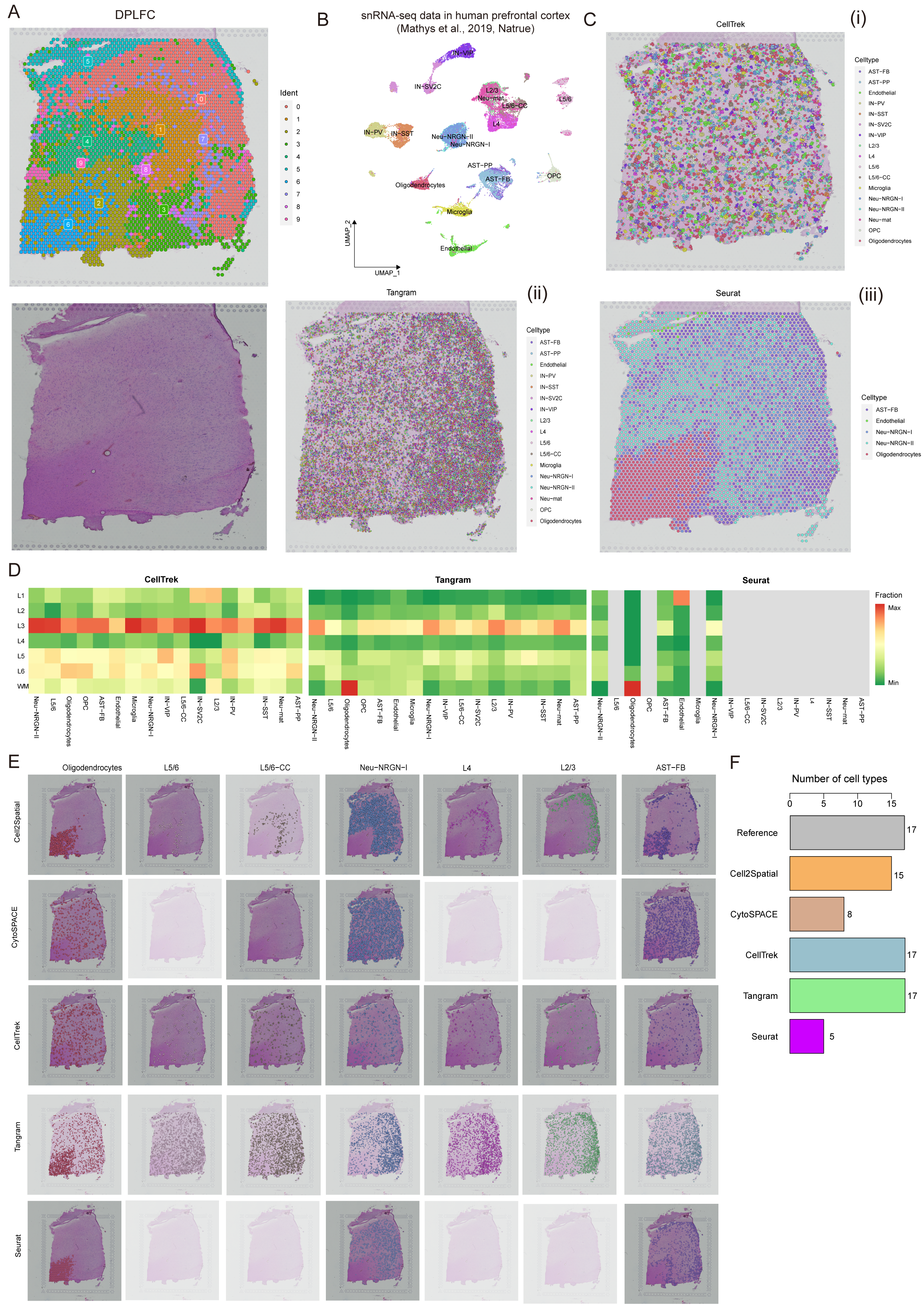

Supplement: S5 Fig — (A) Clustering spots of DPLFC spatial transcriptomics (ST) data. Clusters are marked by color codes (top). Hematoxylin and eosin (H&E) staining image of DLPFC tissue (bottom). (B) Uniform Manifold Approximation and Projection (UMAP) plot showing the single-cell atlas of human prefrontal cortex. Each dot represents an individual cell. Cell types are marked by color codes. (C) Spatial architectures of DLPFC tissue reconstructed using CellTrek (i), Tangram (ii), and Seurat (iii), respectively. Each dot represents an individual cell. Cell types are marked by color codes. (D) Heat map showing the distribution of cell types in anatomical structures of DLPFC. The intensity of the color indicates the cellular fraction within the specific anatomical region. CellTrek (left); Tangram (middle); Seurat (right). (E) Distribution of selected cell types in DLPFC tissue, reconstructed by different mapping tools. Each dot represents one cell. (F) Bar plot showing the number of cell types effectively mapped to spatial locations. “Reference” represents the total number of cell types in the single-cell atlas of the human prefrontal cortex. The underlying data for this figure can be found at https://zenodo.org/records/17212677. (TIF) [file pbio.3003477.s005.tif]

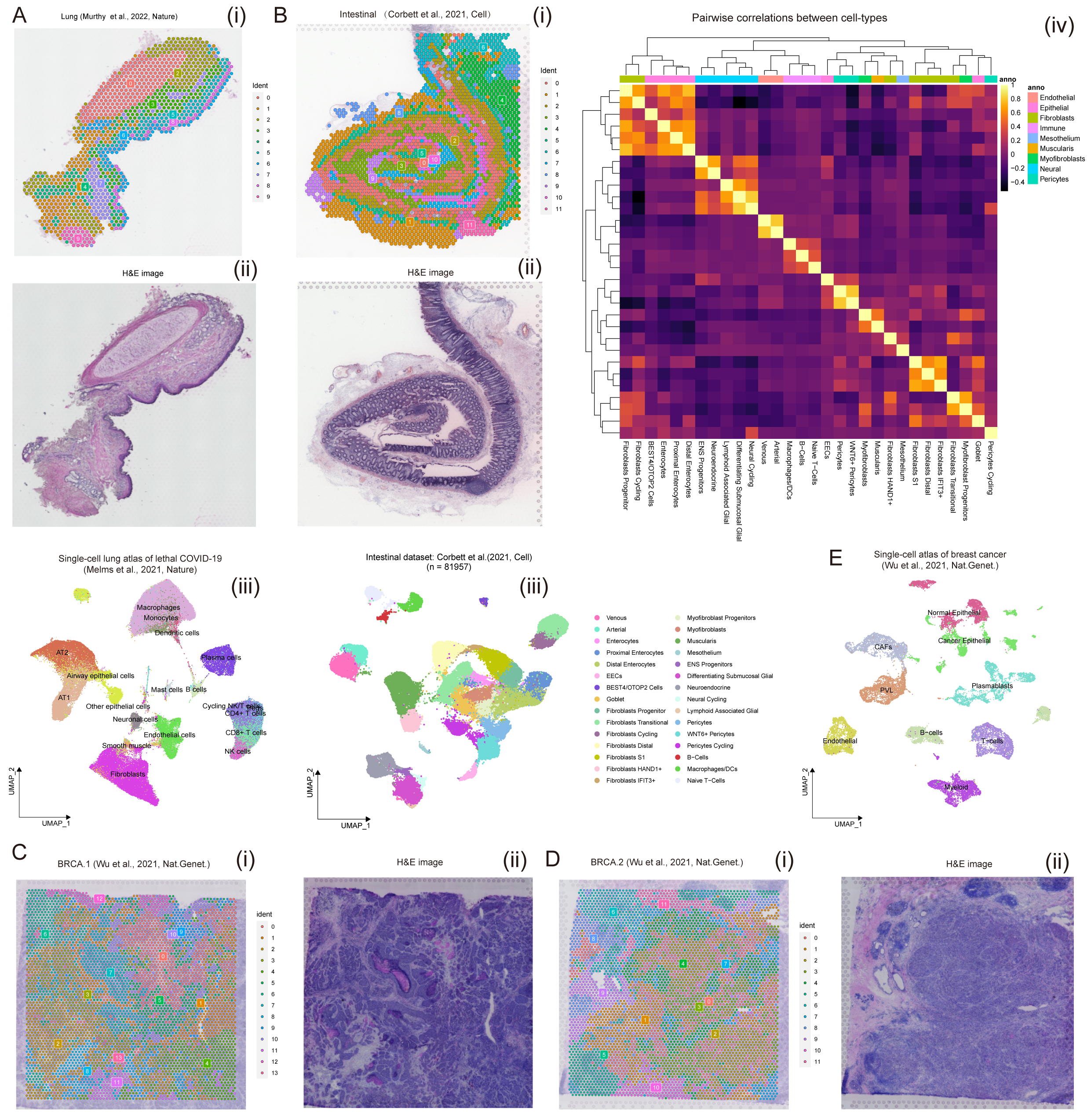

Supplement: S6 Fig — (A) (i) Clustering spots of human lung ST data. (ii) Hematoxylin and eosin (H&E) staining image of human lung tissue. (iii) Uniform Manifold Approximation and Projection (UMAP) showing the single-cell atlas of the human lung under COVID-19 state. (B) (i) Clustering spots of human intestinal ST data. (ii) H&E image of human intestinal tissue. (iii) UMAP plot showing the single-cell atlas of human intestine, annotated based on the marker genes provided by Fawkner-Corbett and colleagues [9]. (iv) Heat map showing pair-wise correlations of annotated cell types. (C, D) Clustering spots of human breast ST data (i); H&E staining image of human breast tissue (ii) [8]. (C) BRCA.1; (D) BRCA.2. (E) UMAP showing the single-cell atlas of human breast cancer patients [8]. Each dot represents an individual cell, and cell types are marked by color codes. The underlying data for this figure can be found at https://zenodo.org/records/17212677. (TIF) [file pbio.3003477.s006.tif]

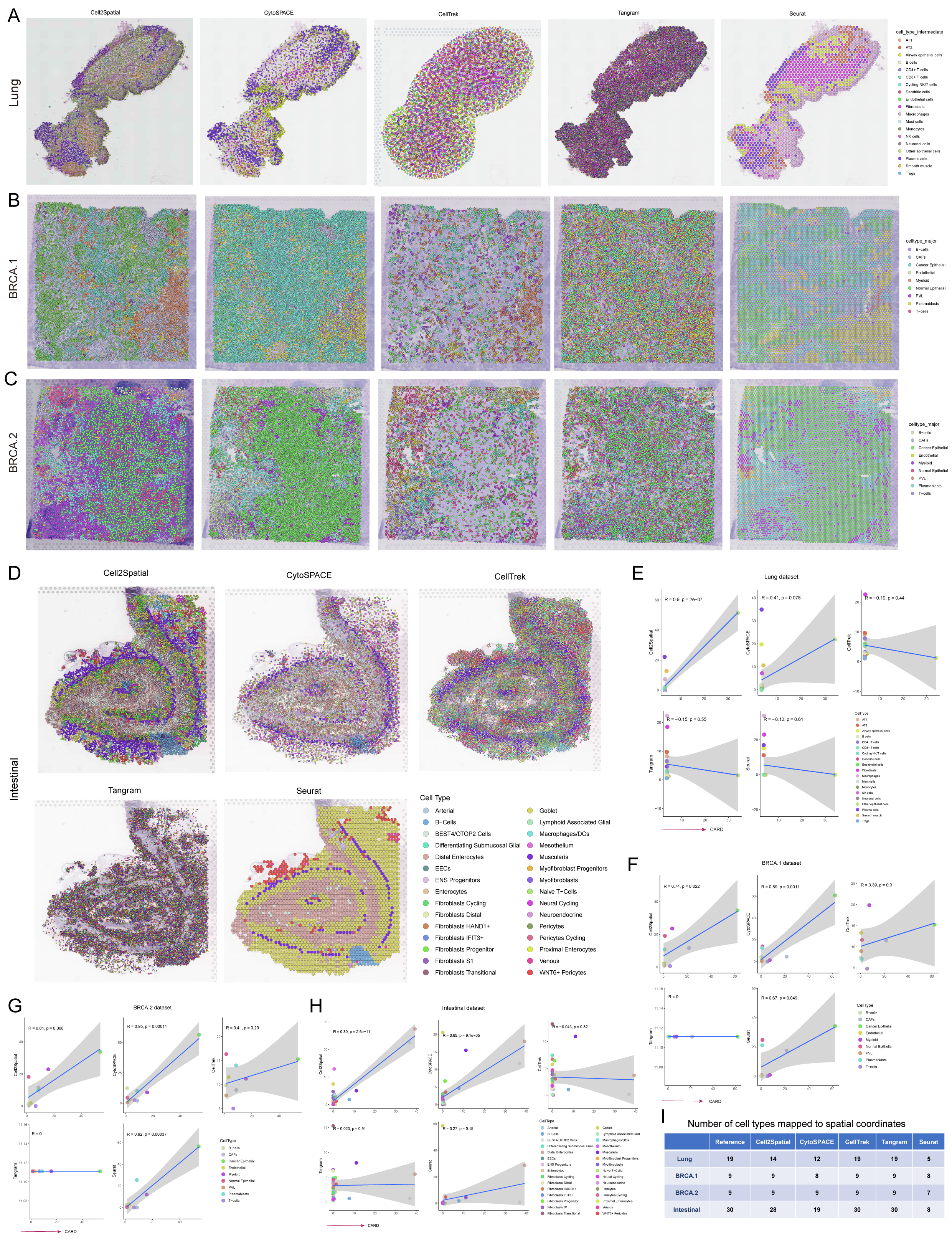

Supplement: S7 Fig — (A–D) Spatial architectures of human Lung (A), Breast (B and C), and Intestinal (D) tissues reconstructed by Cell2Spatial, CytoSPACE, CellTrek, Tangram, and Seurat. Each dot represents an individual cell and cell types are marked by color codes. (E–H) Scatter plots showing the consistency between the cellular proportions in spatial architectures reconstructed with various mapping tools and the cellular compositions predicted by CARD spatial deconvolution tool [11]. The blue line denotes the linear fit, and the shaded area represents the 95% confidence interval. Different colors of points indicate distinct cell types. “R” represents the Pearson correlation coefficient (PCC). P-values were obtained by two-sided t-tests. (E) Lung; (F) BRCA.1; (G) BRCA.2; (H) Intestinal. (I) Table summarizing the number of cell types effectively mapped to spatial locations by each tool. “Reference” represents the total number of cell types in the single-cell atlas of the human tissues. The underlying data for this figure can be found at https://zenodo.org/records/17212677. (TIF) [file pbio.3003477.s007.tif]

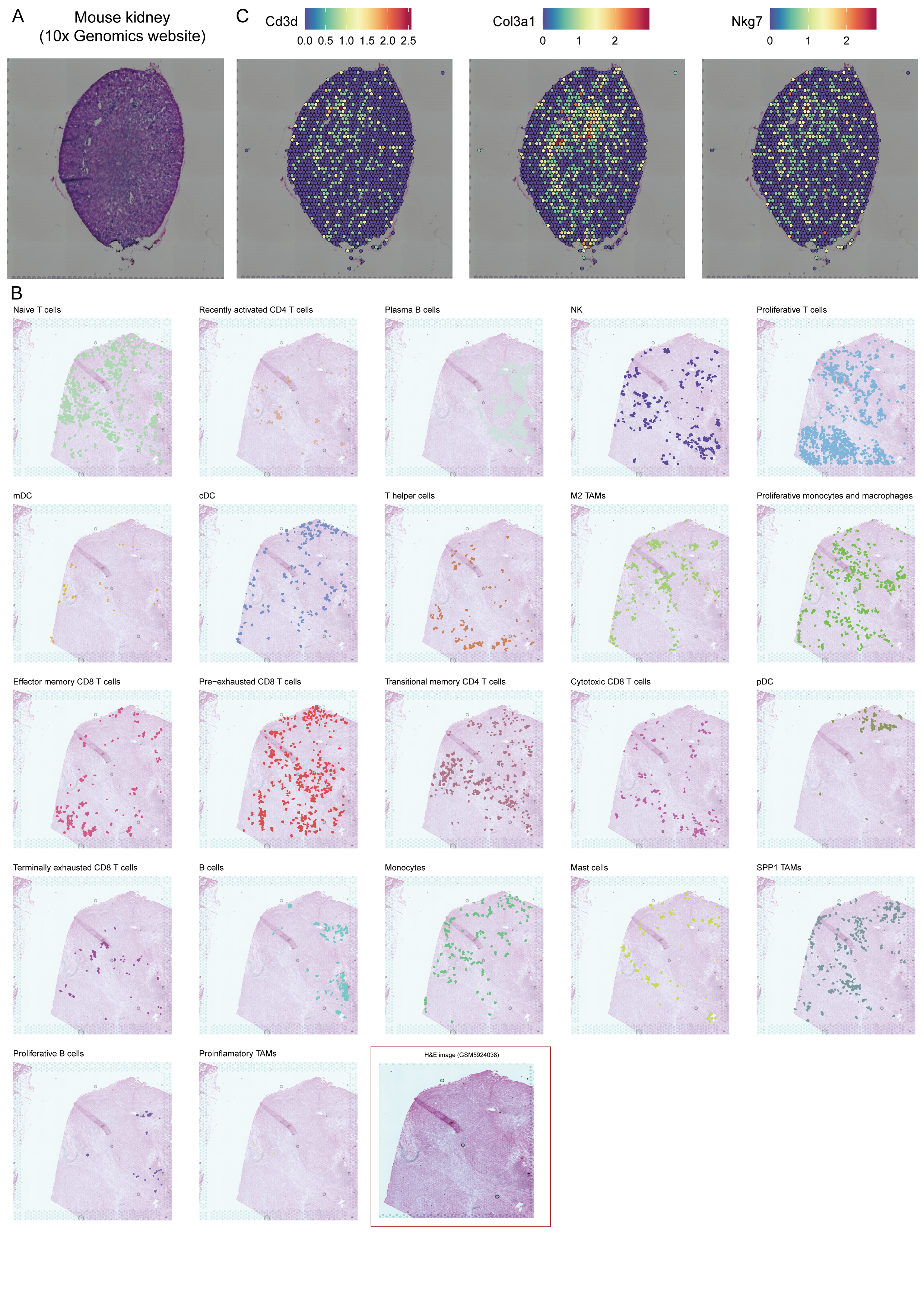

Supplement: S8 Fig — (A) Hematoxylin and eosin (H&E) staining image of mouse kidney tissue. (B) Spatial positioning of immune cell types in RCC tissue sections inferred by Cell2Spatial. The panel with the red border shows the corresponding H&E image of the RCC tissue. (C) Spatial plot showing the expression of selected marker genes in mouse kidney spatial transcriptomics (ST) data. T cell (left); Fibroblasts (Fb, middle); and Natural Killer (NK) cells (right). (TIF) [file pbio.3003477.s008.tif]

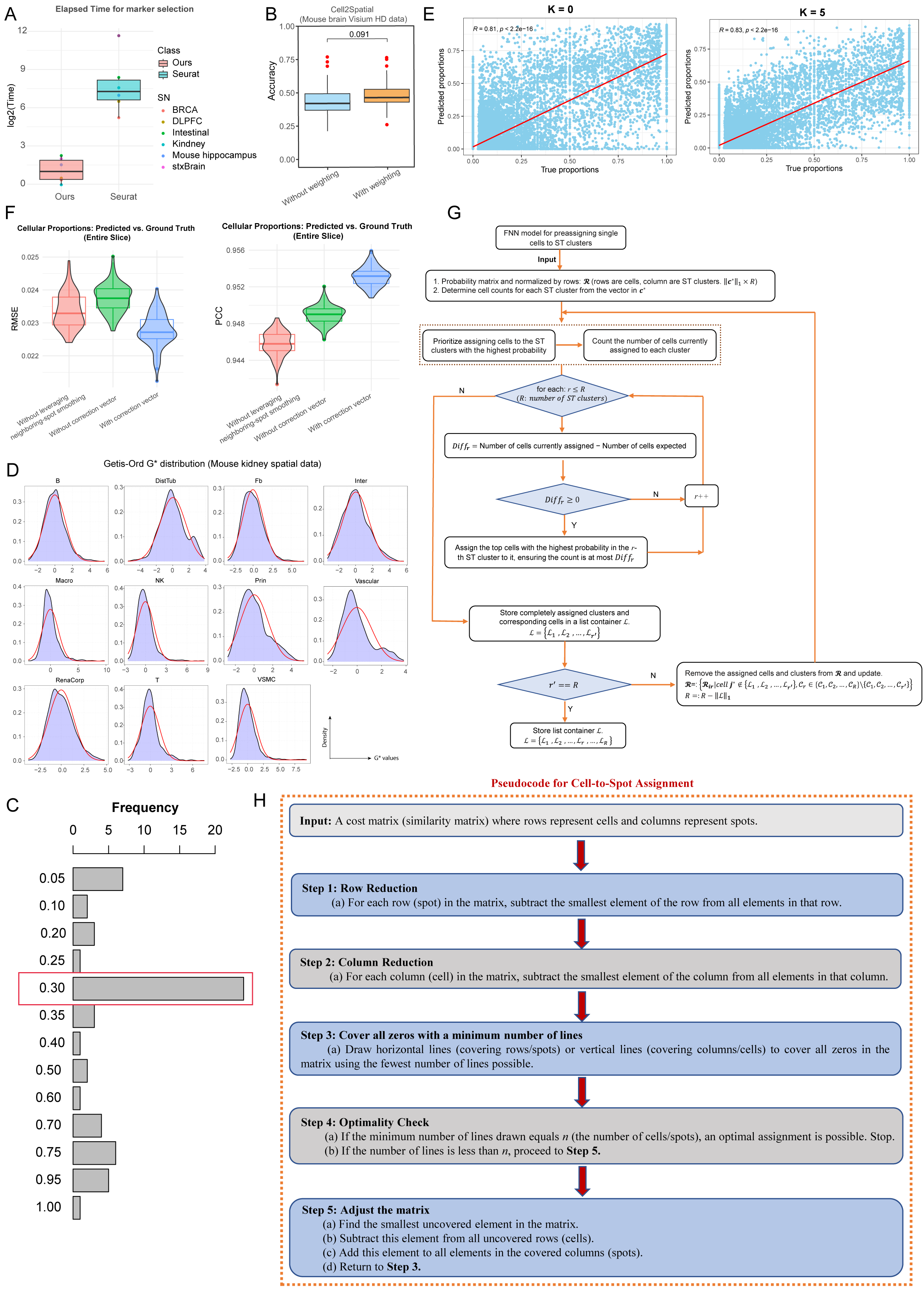

Supplement: S9 Fig — (A) Boxplot showing the distribution of elapsed time for marker selection using Cell2Spatial’s strategy compared with the Wilcoxon test implemented in the Seurat framework. Each point represents one dataset. (B) Boxplot comparing end-to-end mapping accuracy of Cell2Spatial with and without spatial weighting, using mouse brain Visium HD data (8 µm, down-sampled to 50,000 spots). p-value was calculated by the two-sided Wilcoxon test. (C) Bar plot summarizing the frequency with which different quantile cutoffs produced the highest overlap of highly variable genes (HVGs), indicating the most stable cutoff across conditions. To generate these results, Cell2Spatial was applied to map single cells onto mouse kidney spatial transcriptomics (ST) data and reconstruct spatial expression profiles. HVGs were then computed from both reconstructed and original data across gene set sizes ranging from 50 to 3,000, and the consistency of HVG selection was quantified using the Jaccard index. This analysis was repeated across quantile cutoffs from 0.05 to 1 (step = 0.05). (D) Distribution of Getis-Ord G* indices for spatial spots corresponding to each cell type, derived from mouse kidney 10× Visium ST data. The black curve represents the observed Getis-Ord G* distribution, while the red curve shows the fitted normal distribution. (E) Scatter plots showing the predicted cellular compositions of spots by Cell2Spatial. The left panel displays results without smoothing using adjacent spots (k = 0), while the right panel incorporates smoothing with the five nearest neighboring spots (k = 5). True proportions are derived from a simulated ST dataset of the mouse brain. (F) Violin plots combined with boxplots showing the consistency between the estimated overall cellular compositions by Cell2Spatial and the simulated ground truth under different settings. The left panel shows root mean square error (RMSE) values, while the right panel shows Pearson correlation coefficients (PCCs). For each of [file pbio.3003477.s009.tif]
