# Supplementary material for: Cell2Spatial is a computational framework that maps single cells to spatial transcriptomic spots to reconstruct tissue architecture
Source: PLoS Biol. 2025 Nov 17;23(11):e3003477. doi: 10.1371/journal.pbio.3003477 (PMC12638031; doi:10.1371/journal.pbio.3003477)
Supplement: Attachment — Submitted filename: Rebuttal_file_plosbiology_202509.pdf [file pbio.3003477.s021.pdf]

Dear Editor,

We are very grateful to the three reviewers for their positive feedback and thorough comments, which definitely helped us improve our manuscript greatly. We have revised the paper following all of their comments. Below please find the point-by-point response to all the reviewers' comments.

Reviewer1

The authors have successfully addressed all of my concerns. I have no other comments.

Response: We sincerely thank the reviewer for the positive comments and for confirming that all concerns have been addressed. We also greatly appreciate the constructive feedback provided during the review process, which has helped us improve the quality and clarity of the manuscript.

Reviewer2

The revised manuscript is a significant improvement, and the authors have addressed my major concerns. I have some minor comments.

Minor concerns:

1. L23: "Several methods have been publicly disclosed. One common approach, like Seurat [18]" Seurat is an R package with lots of methods. Can you be more specific?

Response: We thank the reviewer for the helpful comment. In the revised manuscript, we have clarified that we are specifically referring to Seurat's anchor-based data integration framework. This framework identifies shared features between reference (SC) and query (ST) datasets and enables the transfer of cell type labels from SC to ST spots **(P3L24-L26)**.

2. L36: "In the case of low-resolution ST data (such as Spatial-based data)" Isn't all ST spatial-based?

Response: We thank the reviewer for pointing this out and apologize for the confusing use of the term "spatial-based". What we intended was specifically "spatial barcoding–based" data. Spatial transcriptomics technologies can be broadly categorized into four strategies: ROI-based (e.g., SMD-seq), image-based (e.g., Xenium), tomography-based (e.g., Geo-seq), and spatial barcoding–based (e.g., 10X Visium). Among these, spatial barcoding–based and image-based approaches are the most widely used in current research.

In the revised version, we have clarified this terminology and explicitly noted that "spatial-based" refers to "spatial barcoding–based" methods (**P3L38**).

3. L68: "Spatial context is incorporated using Seurat's canonical correlation analysis (CCA) [18], which produces a Euclidean distance matrix from the Uniform Manifold Approximation and Projection (UMAP)". Doesn't it use the PCA, not the UMAP?

Response: Thanks for your careful reading and for pointing this out. You are correct that in Seurat, canonical correlation analysis (CCA) is first applied for integration, followed by PCA for dimensional reduction. UMAP is then constructed on the PCA space, and the Euclidean distance matrix is derived from this embedding. Our original description was an oversimplification that may have caused confusion. We have revised the manuscript to clarify this process more accurately **(P4L72-75)**.

4. "For high-resolution ST data, where spots typically capture single cells, it assigns a cell count of 1 per spot.". Both STOMICs and VisiumHD are sub-cellular resolution. Are the spots being binned to be approx. single cell sized? I would also be interested to know what the future development plans are for sub-cellular resolution datasets.

Response: Thanks for your insightful comment. For platforms like VisiumHD with sub-cellular resolution, as you mentioned, we use binning to approximate single-cell resolution. Specifically, we set the *bin.size* parameter in Seurat to aggregate adjacent pixels into virtual spots. In Figure 5G, we set *bin.size* = 8 μm to approximate the size of a single cell. We have updated the manuscript to clarify this process in "Methods" section and prevent confusion regarding high-resolution ST data directly capturing single cells **(P18L596-P19L599)**.

We also appreciate your suggestion regarding future development. In the revised discussion, we highlight two potential directions for handling sub-cellular resolution datasets:

1). Optimizing mapping strategies – Leveraging high-resolution tissue imaging combined with DAPI staining to achieve precise segmentation of single-cell or sub-cellular structures, rather than relying solely on binning.

2). Sub-cellular level modeling – Developing algorithms to directly exploit sub-cellular resolution transcriptomic signals, which would allow us to explore spatial heterogeneity within individual cells, such as transcript distribution between the nucleus and cytoplasm.

These changes are reflected in the latest version of the manuscript **(P14L441-P14L444)**.

5. There are still some improvements that could be made when asserting claims about cell2spatial's performance compared to other tools in the results section of the manuscript. For instance the addition of a % performance difference/increase between tools, or quoting the Cell2Spatial metric and the range in the other tools (as the authors have done in the response to reviewers) could suffice. L110, L128 (Only Jaccard index is quoted for Figure 2?), L153, L164-6 (how compares with the other tools?), L175-7 (other tools?), L194 (other tools?) etc.

Response: We appreciate the reviewer's suggestion to provide more explicit comparative metrics between Cell2Spatial and other tools. In the revised manuscript, we have incorporated detailed performance values and ranges at the indicated sections:

1) L110: We clarified that this part (L110) mainly provides intuitive comparisons, while the quantitative results are presented in Figs. 2D and 2E. At L122–123, we now explicitly report HVG values: Cell2Spatial (0.357), CytoSPACE (0.166), CellTrek (0.221), Tangram (0.145) **(P6L122-L123)**.

2) L128: We included the average of root mean squared error (RMSE) values in text: Cell2Spatial (2.21), CytoSPACE (3.29), CellTrek (4.10), Tangram (8.52), and Seurat (6.38) **(P6L134)**.

3) L153: We revised the text to include distribution ranges for PCC and RMSE values. Cell2Spatial (PCCs: 0.969–0.976; RMSEs: 0.0135–0.0148) outperformed CytoSPACE (PCCs: 0.910–0.974; RMSEs: 0.0134–0.0271), CellTrek (PCCs: 0.777–0.833; RMSEs: 0.0332–0.0402), and Tangram (PCCs: 0.246–0.354; RMSEs: 0.0541–0.0615) **(P7L172–175)**.

4) L164–166: We added explicit cosine similarity ranges in the text: Cell2Spatial (0.787–0.798), CytoSPACE (0.779–0.789), CellTrek (0.753–0.778), and Tangram (0.712–0.736) **(P7L197–198)**.

5) L175–177: We added the average of HVG-conserved scores in the text: Cell2Spatial (0.556), CytoSPACE (0.411), CellTrek (0.361), and Tangram (0.310) **(P8L201–202)**. We also included ranges for Fig. 3F (0.764–0.853; **P8L208**).

Additional updates: We also added quantitative metrics at other relevant lines, e.g., P7L183–186, P8L226–229, and P8L233.

6. L798: Dropbox link? Is there a better repository for this?

Response: Thank you for the helpful suggestion. Unfortunately, Dropbox is inaccessible in China, so we have uploaded the codes and data to several alternative publicly available repositories: 1) Cell2Spatial tool: https://github.com/lihuamei/Cell2Spatial [Zenodo DOI: 10.5281/zenodo.17217934]. 2) Analysis code and source data used for generating figures: https://github.com/lihuamei/Cell2Spatial.Reproduce [Zenodo DOI: 10.5281/zenodo.17212677]. 3) Structured spatial data and corresponding single-cell data: https://drive.google.com/file/d/13D9k1rb47XA7MBbpEcTgwxAlCXNEvdn-/view?usp=drive_link. We hope these links provide easy access to the necessary resources.

Reviewer3

I am pleased to see that the authors have addressed most of the previous concerns. The additional analysis provided by the author strengthens the paper's claims. Overall, I have a positive view of the paper and support its publication. However, I suggest a few minor revisions are needed before the manuscript is finalized.

1. To address the previous comment from Reviewer 1. the authors have incorporated a comprehensive benchmarking study in their revised manuscript, citing Li et al., 2022. They have used their own simulated spatial transcriptomics (ST) datasets to evaluate the performance of Cell2Spatial and other tools. While this is a valuable exercise, the Li et al. paper provides its own set of benchmark datasets (already published). To further validate their claims of superior performance and provide a more direct comparison to the published literature, it would be highly informative to simply apply Cell2Spatial on those datasets and see how it performs on the publicly available benchmark datasets from the Li et al. study. I would recommend that the authors run their tool on these datasets and include the results in the manuscript.

Response: We thank the reviewer for this constructive suggestion. Following the reviewer's advice, we applied Cell2Spatial to the 32 benchmark single-cell and simulated ST datasets from Li et al., 2022. These datasets include human brain, liver, lung, kidney, pancreas, and mouse pancreas and trachea, with a median of 8 cell types per dataset (ranging from 6 to 128). Each simulated ST dataset contains 1,000 spots with known cellular compositions and cell counts.

On these datasets, Cell2Spatial demonstrated strong performance in reconstructing spot-level cell type compositions **(Fig. 3B)**. The average Pearson correlation coefficient (PCC) between predicted and true cell type proportions was 0.784 (range: 0.427–0.964), ranking second among all evaluated tools, just after Cell2location (range: 0.620-0.942). The average root mean square error (RMSE) was 0.114 (range: 0.027–0.220), ranking fifth overall. While Cell2location achieved the best performance on both PCC and RMSE, Cell2Spatial showed competitive accuracy.

We also compared Cell2Spatial with CytoSPACE for predicting spot-level cell counts, using the ground truth cell counts provided in the datasets **(Fig. 3I)**. Cell2Spatial achieved an average PCC of 0.761 (range: 0.506–0.903), consistently outperforming CytoSPACE (mean PCC = 0.700; range: 0.480–0.898). The median RMSE for Cell2Spatial was 2.09 (range: 1.64–3.85), lower than CytoSPACE (median = 2.55; range: 1.94–3.83). This improvement was statistically significant (p-value < 0.01; Wilcoxon test).

These changes are reflected in the latest version of the manuscript **(Figs. 3B, and I; P7L177-L188; P8L224-P8L229)**.

2. Figure 2H, Consistency Result: The box plot shows the Jaccard index for single-cell mapping accuracy at different noise levels. Firstly, it is counterintuitive that for some tools, like CellTrek, the consistency seems to increase as the noise level increases. This could suggest that the consistency results are also subject to a high degree of randomness and using only one dataset is not enough for a consistent comparison. Additionally, the Jaccard index values for all tools are quite low, with the highest mean index being less than 0.20. The author should provide some comments/discussions on these phenomena.

Response: We thank the reviewer for the valuable comment. In response, we added two additional replicates at each noise level in the mouse brain simulations **(Fig. 2H)**. While CellTrek initially appeared to improve as noise increased, the replicates show that this pattern is not consistent. In the second

replicate, the Jaccard indices were 0.203 at 0% noise, 0.191 at 5% noise, and 0.185 at 20% noise; in the third replicate, the corresponding values were 0.198, 0.197, and 0.187. Similar trends were observed with other tools, such as Cell2Spatial, CytoSPACE, and Tangram, where Jaccard indices decreased with higher noise levels. This suggests that the initial improvement seen with CellTrek was likely due to random variation, rather than a real effect.

We also agree with the reviewer's point about the low Jaccard values (<0.20). This is likely because current mapping methods focus on features that separate different cell types, which have limited ability to distinguish cells within the same type. As a result, these tools are better at reconstructing cell-type distributions rather than pinpointing the exact spatial origin of individual cells. We appreciate the reviewer's insights, which will guide our future research.

These changes are reflected in the latest version of the manuscript **(Fig. 2H; P6L148-L157; P13L438-P14L441-L442)**.

3. Figure 3H, Lower-Left Panel: The lower-left panel of Figure 3H, which displays the cell counts in a whole mouse brain ST slice using Cell2Spatial, shows a large contiguous area with the same cell count. The authors should provide an explanation for this phenomenon and exclude those spots from downstream analysis if appropriate.

Response: We thank the reviewer for the valuable comment. To clarify, the lower-left panel of Figure 3H in the manuscript actually shows nuclei counts derived from the DAPI image using Squidpy, while the middle panel displays cell counts predicted by Cell2Spatial. Upon review, we found that the large contiguous area with identical counts was caused by an incorrect selection of the channel (channel=0) from the H&E image when calculating nuclei counts with Squidpy tool (Palla et al.; *Nature methods*; 2022).

After correcting the channel selection to channel=2, this issue was resolved. Additionally, we re-ran the cell counts inferred by Cell2Spatial and CytoSPACE, as resetting the maximum and median number of cells in the spots impacted predictions. Following these corrections, we updated **Figures 3J** (bottom panel) and **3L**. The revised results show better alignment between Cell2Spatial predicted and DAPI-based cell counts (R = 0.49), outperforming CytoSPACE (R = 0.35).

These changes are reflected in the latest version of the manuscript **(Fig. 3J (bottom panel), 3L; P8L233)**.

4. Typo in Figure 1: In the upper-right portion of Figure 1, within the "Single-cell RNA-seq (SC) data" section, there is a typo in the legend. The "Cell Type" list shows "Type4" twice. This should be corrected for clarity.

Response: We thank the reviewer for pointing this out and apologize for the oversight. In the latest version, we have corrected the typo in Figure 1 and replaced the generic labels "Type 1–4" with specific cell type names (e.g., T cell, NK cell, B cell, Monocytes, Fibroblasts) to enhance clarity and interpretability **(Fig. 1)**.
